# High-Resolution Satellite Imagery Is an Important yet Underutilized Resource in Conservation Biology

**DOI:** 10.1371/journal.pone.0086908

**Published:** 2014-01-23

**Authors:** Sarah A. Boyle, Christina M. Kennedy, Julio Torres, Karen Colman, Pastor E. Pérez-Estigarribia, Noé U. de la Sancha

**Affiliations:** 1 Department of Biology, Rhodes College, Memphis, Tennessee, United States of America; 2 Development by Design Program, The Nature Conservancy, Fort Collins, Colorado, United States of America; 3 Unidad de Investigación Sistemática, Diversidad y Evolución, Centro Nacional Patagónico, Puerto Madryn, Chubut, Argentina; 4 Dirección de Vida Silvestre, Secretaría del Ambiente, Asunción, Paraguay; 5 Programa de Magister en Ciencias, Mención en Zoología, Universidad de Concepción, Concepción, Chile; 6 Science and Education, The Field Museum of Natural History, Chicago, Illinois, United States of America; Institute of Ecology, Germany

## Abstract

Technological advances and increasing availability of high-resolution satellite imagery offer the potential for more accurate land cover classifications and pattern analyses, which could greatly improve the detection and quantification of land cover change for conservation. Such remotely-sensed products, however, are often expensive and difficult to acquire, which prohibits or reduces their use. We tested whether imagery of high spatial resolution (≤5 m) differs from lower-resolution imagery (≥30 m) in performance and extent of use for conservation applications. To assess performance, we classified land cover in a heterogeneous region of Interior Atlantic Forest in Paraguay, which has undergone recent and dramatic human-induced habitat loss and fragmentation. We used 4 m multispectral IKONOS and 30 m multispectral Landsat imagery and determined the extent to which resolution influenced the delineation of land cover classes and patch-level metrics. Higher-resolution imagery more accurately delineated cover classes, identified smaller patches, retained patch shape, and detected narrower, linear patches. To assess extent of use, we surveyed three conservation journals (*Biological Conservation*, *Biotropica*, *Conservation Biology*) and found limited application of high-resolution imagery in research, with only 26.8% of land cover studies analyzing satellite imagery, and of these studies only 10.4% used imagery ≤5 m resolution. Our results suggest that high-resolution imagery is warranted yet under-utilized in conservation research, but is needed to adequately monitor and evaluate forest loss and conversion, and to delineate potentially important stepping-stone fragments that may serve as corridors in a human-modified landscape. Greater access to low-cost, multiband, high-resolution satellite imagery would therefore greatly facilitate conservation management and decision-making.

## Introduction

Since the 1972 launch of the Earth Resources Technology Satellite (renamed Landsat 1), remotely sensed imagery has been increasingly used to monitor Earth’s ecosystems [1]–[5] by quantifying land cover change [6], deforestation [7], carbon stocks and emissions [8], habitat degradation and disease [9], [10], species diversity [11], [12], invasive species [13], habitat suitability [14], and species populations [15]. Satellite imagery products, however, vary in their spatial and spectral resolution, geographic and temporal coverage, cloud cover, security regulations, and price [4], [6], [16], [17]–variables that can hamper their consistent application in conservation. For example, not all areas of the globe have equal access to high-resolution data, with tropical areas having the least coverage available [18].

Satellite imagery employed in conservation research ranges from 1000 m to <1 m in resolution [4], [5]. Global- to biome-scale monitoring of forest clearing is often conducted at spatial resolutions of 250–1000 m [5], [6]. Landsat imagery (30 m multispectral resolution) has been integral in scientific research since 1972 [19], particularly in mapping and assessments of land cover change [20], and it is currently available at no cost [21]. High-resolution imagery (e.g., IKONOS and QuickBird at ≤5 m resolution) is typically used to map regional-to-local areas and species, and to inform land cover classifications derived from coarser imagery; but often such imagery is expensive and cost-prohibitive [5]. One exception is the free, high-resolution imagery provided via Google Earth that is increasingly being used in scientific research [22], [23], can aid in the selection of field sampling locations [10], and can be used as training samples for classification [24]. Imagery analysis based on Google Earth images, however, is limited as the different satellite bands are not available for manipulation by the user.

Previous comparisons of land cover classifications based on imagery of varying spatial resolutions (i.e., IKONOS, Landsat) have revealed mixed results, with one type of satellite imagery failing to consistently perform best across different studies and systems [25]–[29]. Although high-resolution imagery often outperforms lower-resolution imagery in capturing small habitat patches [27], [30], it can produce more canopy shadow [31], and complicate multi-image comparisons and processing [9].

We assert that conservation research would benefit from a better understanding of satellite imagery performance. The ability to detect forest disturbance and degradation (e.g. as a result of selective logging) with satellite imagery varies greatly [32], [33], and missed detection can lead to false conclusions that natural systems are intact when in fact they have undergone high levels of disturbance [34], [35]. Furthermore, readily available vegetation maps and data sets often mosaic multiple imagery from various time periods [5], which may lead to difficulties in comparing land cover change. Articles focusing on the applications of satellite imagery and remote sensing are often published in remote-sensing, physical geography, informatics, and ecology journals (see reviews by [2], [4], [36]–[44]), which may limit the extent to which conservation biologists encounter these topics. The increasing availability of high-resolution imagery has the potential to provide more accurate detection and quantification of habitat conversion, degradation and fragmentation [35], [37], but we suspect such imagery are not being taken advantage of by conservation biologists, even though their usage can result in more accurate habitat and ecosystem assessments. To test this assertion, we compared land cover classifications in the Interior Atlantic Forest of Paraguay (an area of current conservation concern due to recent and extensive forest loss [20]), using 4 m multispectral IKONOS and 30 m multispectral Landsat imagery and evaluated whether imagery resolution significantly influences delineation of land cover classes and fragmentation metrics of forest patches. We then reviewed current literature from three conservation journals to determine the extent and the type of usage of satellite imagery for conservation purposes. Our findings have important implications regarding the utility of higher-resolution imagery to monitor habitat change, and to potentially model connectivity for species within a fragmented landscape.

## Materials and Methods

### Imagery Analysis of Interior Atlantic Forest

**Figure 1:**
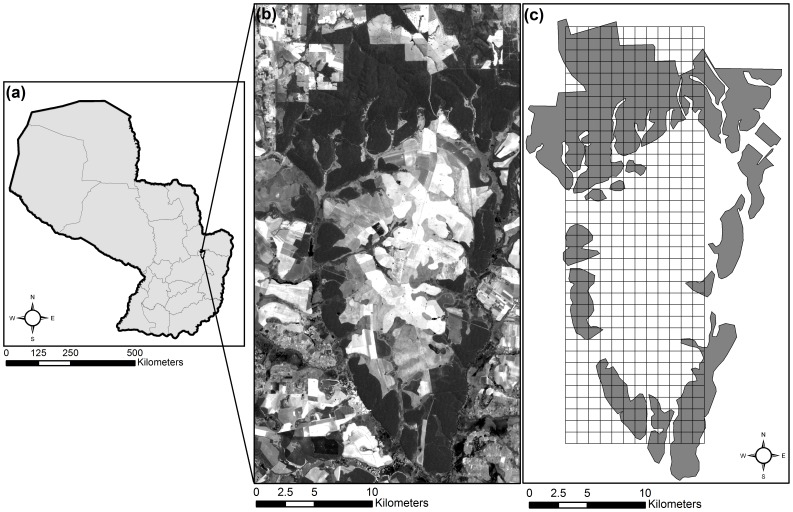
Study location. The site (a) is located in eastern Paraguay at a private forest reserve, Reserva Morombi. Forest patches (b) are shown in dark, surrounded by a heterogeneous matrix, in the grey-scale Landsat TM image. Comparison of IKONOS- and Landsat-based classification extended across 43,200 ha, and was based on subsampling (c) using 432 1 km×1 km grid cells.

The Atlantic Forest of South America is one of the major global “hotspots” for biodiversity due to its large number of endemic species and its dramatic decrease in size due to human-induced habitat loss and fragmentation [45], [46]. Paraguay has lost 85 percent of its Atlantic Forest during the past 60 years [47]–[49], and it is one of the least-studied countries in South America [50], with little known regarding lost species and those in imminent danger of disappearing. This pattern of deforestation has continued in Paraguay to present [51]. We assessed a heterogeneous landscape directly surrounding and including Reserva Natural Privada Morombi (centroid coordinates: 24° 39′ S, 55° 22′ W), a private 25,000 ha reserve in the Departments of Canindeyú and Caaguazú, Paraguay (Fig. 1). Reserva Morombi consists of remnant patches of semi-deciduous Interior Atlantic Forest, surrounded by a matrix of agriculture (pasture and cropland), wetlands, and grasslands.

We obtained cloud-free, geometrically corrected, and orthorectified IKONOS (4 m multispectral resolution) and Landsat (30 m multispectral resolution) imagery for the study region. Both imagery types spanned a 43,200 ha area that contained forest patches within the reserve, as well as the surrounding heterogeneous matrix. We selected Landsat imagery because it is readily and freely available, and frequently used for land cover classification [52]; we selected IKONOS imagery because it is often used for vegetation assessment [5]. Dates of imagery were paired relatively close in time (September 2008 and July 2009 for IKONOS and Landsat, respectively) to help ensure consistency in cover classes and phenology; we were, however, limited by the available date for IKONOS imagery and the inability to use Landsat imagery from September 2008 due to sensor malfunction (ETM+ on Landsat 7; [16]) and cloud coverage (Landsat 5). The differences in imagery dates (2008 and 2009) and date of field data collection (2010) did not impact our results, given that there were no major changes in land cover from 2008 to 2010 in the study area (as referenced from Landsat images during this time period, and likely a result of the protection status of the study area).

We collected 250 ground-truth points in the study area in June 2010 to serve as training samples for the classification; these points were taken throughout the private reserve. Using ERDAS Imagine 10, we drew polygons around each point and assigned the polygon to a land cover class. Training samples (number of locations and number of pixels) were distributed evenly across classes, with the exception of water because this class was limited in its spatial distribution across the study area (<1% of study area). We used the same 250 locations for both IKONOS and Landsat classifications. We used transformed divergence [53], to validate the suitability of these training classes; all comparisons met the standards with values >1700. We conducted pixel-based, supervised classifications of both images based on the maximum likelihood algorithm, and delineated six general cover classes (forest, wetlands, grasslands, agriculture, cleared, and water; definitions of land cover classes available in Table 1). The agricultural class originally consisted of two separate classes of crops and pasture, but during post-classification processing these classes were merged into a single ‘agriculture’ class to facilitate more general comparisons with other land cover classes, as is commonly done in conservation studies [54], [55]. We measured classification accuracy by randomly selecting 204 point locations (evenly distributed among the six classes) from a 1 m resolution panchromatic IKONOS image from the study area. We calculated accuracy and kappa statistics (*k*) for the overall classification and for each of the six land cover classes [56], determining the accuracy of the 204 locations in both the IKONOS and Landsat classified data sets.

**Table 1:** Description of land cover classes delineated in this study.

Class	Description
Forest	Semi-deciduous trees >2 m in height
Agriculture	Crop (e.g., maize, soybean, wheat) fields and cattle pasture^a^
Cleared	Barren ground lacking vegetative cover, including roads, and infrastructure such as houses and barns
Wetland	75–100% herbaceous vegetation cover and water-saturated soil
Grassland	75–100% perennial grasses not used for cattle grazing
Water	Bodies of water, including lakes and ponds

a Agricultural components (i.e. crop fields, pasture) were combined into one class for general comparisons across the broader land cover classes.

We converted raster data to vector data in ArcGIS 9.3 to calculate patch-level metrics. Continuous pixels were combined into a single vector patch, allowing for the quick delineation of number of patches and their sizes. We converted raster data to vector data instead of using object-based landscape analysis [57], [58], due to the ease of working with vector data in ArcGIS 9.3, software commonly used by authors in our literature review. Due to differences in pixel size we expected the high-resolution classification to result in a greater number of patches; therefore, we compared metrics based on all patches as well as only patches ≥0.5 ha, following previous fragmentation studies [59], [60], that used a 0.5 ha minimum patch size.

A grid consisting of 36 rows and 12 columns (432 1-km×1-km grid cells) was overlaid on both IKONOS- and Landsat-derived classifications (Fig. 1c). For each grid cell, we calculated the area (in ha) and number of patches occupied by each of the six land cover classes. We then tested whether area and/or number of patches significantly differed between the two imagery types based on Multivariable Analysis of Variance (MANOVA) with the six land cover types as variables (α = 0.05). Post-hoc Tukey tests were used to test significant differences between the imagery types (IKONOS and Landsat) for individual land cover classes (with familywise α = 0.05).

We calculated patch-level fragmentation metrics (i.e. patch area, patch edge, shape index, perimeter-area ratio) using Patch Analyst 5.0 [61], based on all patches and patches ≥0.5 ha. We calculated the distance from each forest patch’s centroid to the closest patch’s centroid (nearest neighbor) and the mean distance from a target patch centroid to all other centroids using Hawth’s Tools for ArcGIS [62], to test for differences in forest patch configuration. We used Euclidean nearest neighbor distances because they are the most widely used connectivity metrics [63]. Differences between IKONOS and Landsat imagery classifications in patch-level and connectivity metrics were determined by t-tests.

Linear habitat features, such as riparian corridors, have been found to be important conduits for species movement, thus, facilitating landscape-level connectivity [64]. To determine the effects of imagery resolution on the detection of such narrow features, we chose 40 random locations in forest widths ranging from 3.5–100 m from a 1 m panchromatic IKONOS image. We then tested for differences between IKONOS and Landsat in detectability of these linear habitat features using a paired t-test, and the correlation between patch width and its detection using Pearson’s correlations. All statistical analyses were conducted in Matlab 6.5.01 (The MathWorks, Inc., 2006).

### Extent of Imagery Use by Conservation Biologists

We conducted a literature review of three top-tier conservation journals (*Biological Conservation*, *Biotropica*, and *Conservation Biology*) to assess the extent to which satellite imagery is currently utilized in conservation research. We reviewed all articles published in these three journals for the past two completed calendar years (January 2011 to December 2012), examining the methods to determine if satellite imagery was used in analyses. We did not include book reviews, editorials, or letters commenting on previous articles, and we limited our analysis to studies examining terrestrial (non-marine) patterns. For those studies utilizing remotely sensed data, we documented the satellite type (e.g. IKONOS, Landsat), the spatial resolution of the imagery, the type of analysis (e.g. land cover change, patch-based metrics), and the scale of analysis (i.e. global, continental, country, state, local).

## Results

### Imagery Classification Performance

**Figure 2:**
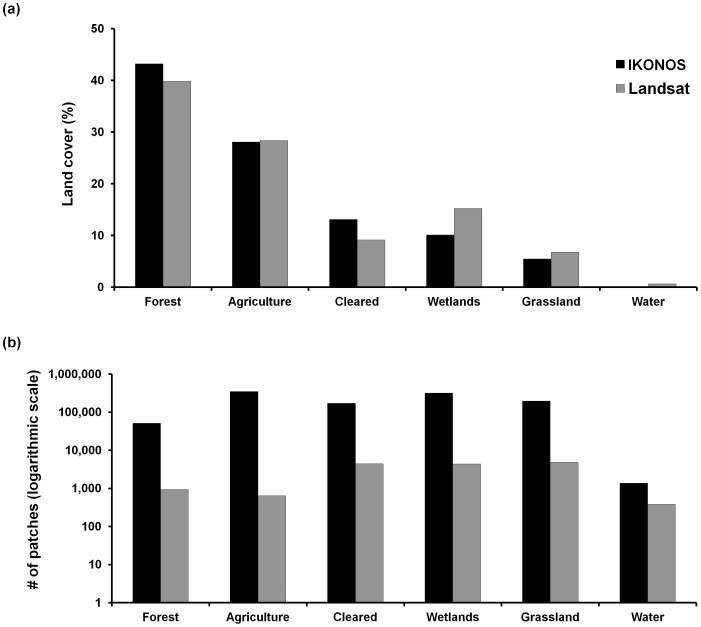
Comparison of imagery performance. IKONOS and Landsat imagery classifications significantly differed in (a) percent land cover and (b) total number of patches for the six land cover classes found in the study area in Paraguay.

Overall classification accuracy was 90.3% for IKONOS (*k* = 0.89) and 87.9% for Landsat (*k* = 0.86). For both IKONOS and Landsat, kappa statistics were lowest for the wetland class (*k* = 0.68 and *k* = 0.54, respectively). The sample within a section of Reserva Morombi and its environs were dominated by forest, followed by agriculture, based on both IKONOS and Landsat classifications (Fig. 2a). Land cover classifications based on the two satellites, however, significantly differed in area of the six land cover classes (MANOVA, *F*_6, 857_ = 5.53, *p*<0.001), with significant differences in cleared (pairwise Tukey test: *F*_1, 862_ = 3.88, *p* = 0.006), water (*F*_1, 862_ = 5.04, *p*<0.001), and wetland (*F*_1, 862_ = 6.21, *p*<0.001) classes. Land cover based on IKONOS resulted in a greater number of patches overall for all six cover types (*F*_6, 857_ = 160.69, *p*<0.001; Fig. 2b) and for all six classes (Tukey test: *p*<0.01 for all pairwise comparisons) relative to Landsat.

**Figure 3:**
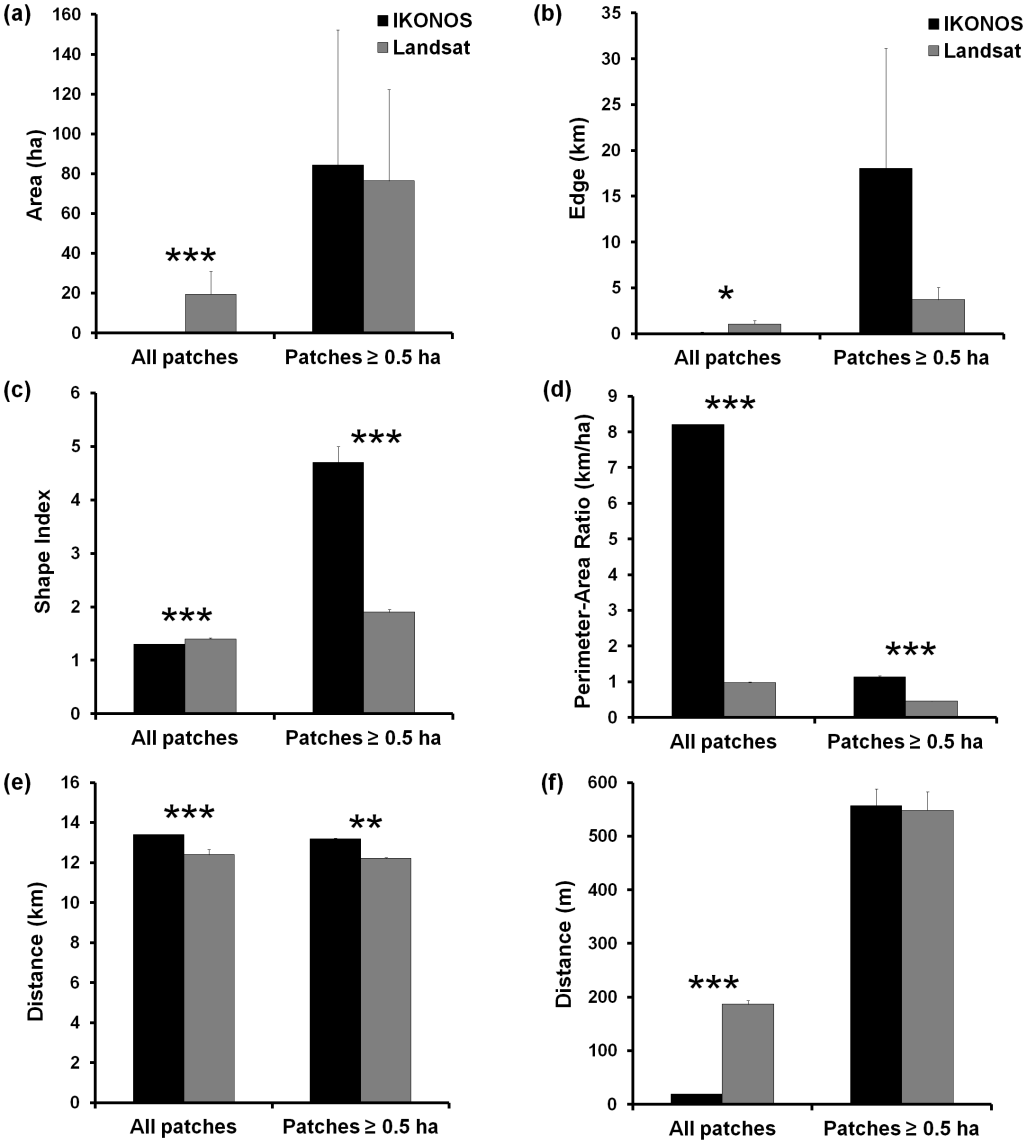
Patch metrics varied with imagery type. IKONOS and Landsat classifications significantly differed in patch metrics for all forest patches and those ≥0.5 ha in (a) patch area; (b) patch edge; (c) shape index; (d) perimeter-area ratio; (e) mean distance from patch centroid to all other patch centroids; and (f) distance from patch centroid to the closest patch’s centroid. Asterisks (*, **, ***) indicate significant differences at *p*≤0.05, 0.01, 0.001, respectively; with df = 52,168 and df = 464 for all t-tests using data from all patches and from patches ≥0.50 ha, respectively. Error bars represent one standard error.

In total area, forest comprised 43.2% of the IKONOS image and 39.8% of the Landsat image, with a difference of 1473.6 ha. Although total forest area did not differ greatly between the two classifications, there were significant differences between IKONOS and Landsat in all six of the patch-level metrics we tested when analysis included all forest patches (Fig. 3): patch area (*t* = −6.94, df = 52,168, *p*<0.001), patch edge (*t* = −2.17, df = 52,168, *p = *0.030), shape index (*t* = −6.20, df = 52,168, *p*<0.001), perimeter-area ratio (*t = *99.34, df = 52,168, *p*<0.001), mean distance from patch centroid to all other patch centroids (*t = *10.34, df = 52,168, *p*<0.001), and distance from patch centroid to closest patch’s centroid (*t* = −137.23, df = 52,168, *p*<0.001). When we included only forest patches ≥0.5 ha, significant differences existed between IKONOS and Landsat in three of the six patch-level metrics: shape index (*t = *8.180, df = 464, *p*<0.001), perimeter-area index (*t = *15.71, df = 464, *p*<0.001), and mean distance from patch centroid to all other patch centroids (*t = *2.90, df = 464, *p = *0.004).

**Figure 4:**
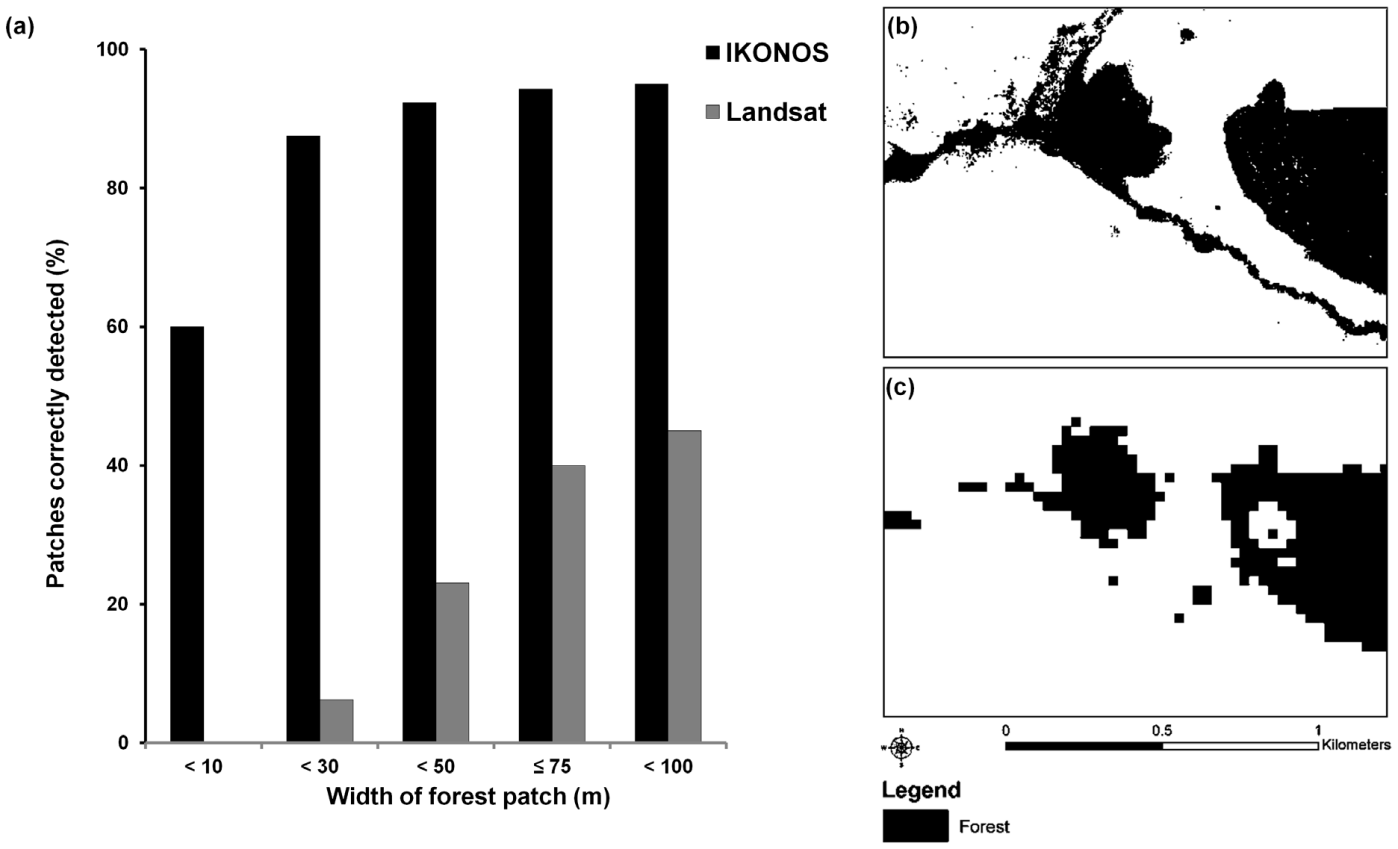
Detection of linear forest features varied between IKONOS (4 m resolution) and Landsat (30 m resolution). IKONOS correctly identified more narrow forest fragments than Landsat (a) as evident in one example from the study area with (b) IKONOS preserving small forest fragments and forested corridors better than (c) Landsat.

Although mean patch size was smaller with IKONOS imagery, IKONOS had the largest patch (15,565 ha vs. 10,562 ha with Landsat) and delineated 55 times more forest patches than did Landsat, most of which were <0.5 ha. IKONOS correctly detected 95% of linear forest fragments 3.5 m –100 m in width, while Landsat detected only 45% of these same patches (Fig. 4a). IKONOS successfully detected 100% of forest fragments >6 m in width, while Landsat only correctly detected 70.8% of the fragments >30 m wide; even when fragments were >50 m, Landsat correctly identified only 85.7% of the fragments. This difference in detectability of narrow forest features was significant (*t* = 6.25, df = 39, *p*<0.001), and was correlated with the width of the forest fragment for both IKONOS (*Ρ* = −0.32, *p* = 0.046) and Landsat (*Ρ* = −0.68, *p*<0.001). For example, unlike in the IKONOS classification (Fig. 4b), long, linear features, such as a narrow forest corridor of 9–64 m in width, was primarily missed with Landsat (Fig. 4c). Only ∼30% of patches (72 of 229 IKONOS patches and 237 Landsat patches) matched one-to-one between the two classifications.

### Satellite Imagery in Conservation Research

**Figure 5:**
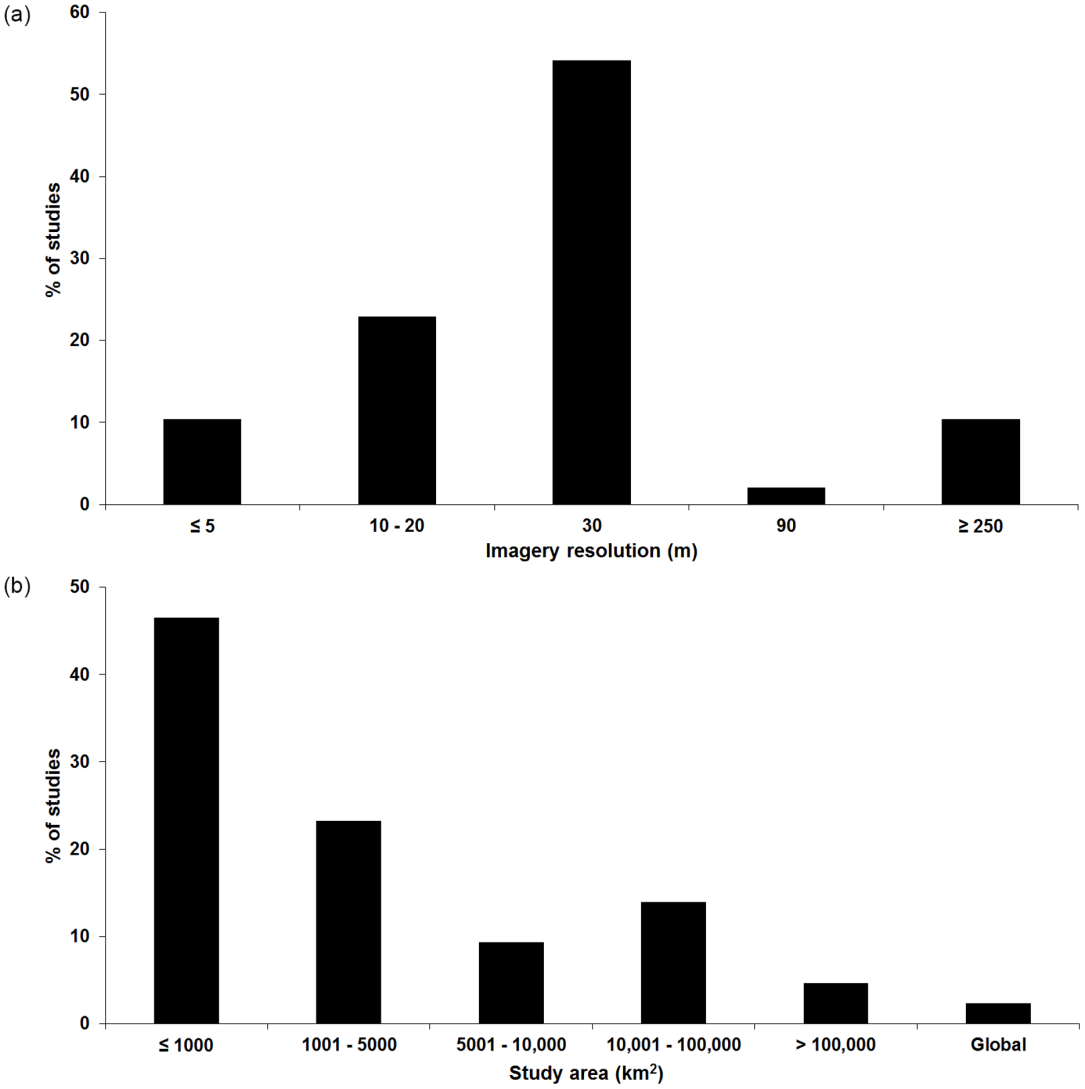
Limited use of high-resolution imagery for conservation. Out of 1064 articles in *Conservation Biology*, *Biological Conservation*, and *Biotropica* (2011–2012), 157 utilized primary satellite imagery and analyzed land cover predominantly based on (a) satellite imagery of 30 m resolution and (b) quantified geographic areas ≤1000 km^2^ (equivalent to ≤100,000 ha).

1064 articles were reviewed in the three target conservation journals (*Biological Conservation*, *Biotropica*, *Conservation Biology*), of which 14.8% used land cover data in analyses. Of these 157 articles using land cover data, 26.8% used primary satellite imagery, while 73.2% used paper maps, aerial photos, Google Earth, or land cover data previously published by other authors. Of the 42 studies that analyzed satellite imagery, mean resolution was 84.4 m, with 66.7% and 10.4% using imagery ≥30 m and ≤5 m, respectively (Fig. 5a). Landsat was the most common satellite (51.0% of articles), followed by SPOT (14.3%), and Terra/MODIS (10.2%); additional data sources (e.g. IKONOS, QuickBird, Lidar, CBERS, ASTER; Table 2) each were represented in <5% of the articles. Most studies (67.4%) analyzed a geographic area of 500,000 ha or smaller (Fig. 5b).

**Table 2:** Characteristics of satellites and their sensors used in studies classifying land cover in manuscripts published in *Conservation Biology*, *Biological Conservation*, and *Biotropica* 2011–2012.

Satellite/Sensor	First launch	Resolution (m)^a^	Current status
Advanced Land Observation Satellite (ALOS)/Advanced Visible andNear Infrared Radiometer type 2 (AVNIR-2)	2006	10	Retired
China-Brazil Earth Resources Satellite (CBERS)	1999	20–80	Active
Earth-Observing 1/Advanced Land Imager (ALI)	2000	30	Active
European Remote-Sensing Satellite (ERS)	1991	25	Retired
IKONOS	1999	4	Active
Landsat/TM, ETM+/OLI/TIRS	1972	80 (1970s); 30 (1982– present)	Active
Light Detection and Ranging (Lidar)^b^	Multiple systems exist	variable	Active
QuickBird	2001	2.4	Active
Systėme Pour l’Observation de la Terre (SPOT)	1986	8–20	Active
Shuttle Radar Topography Mission (SRTM)^b^	2000	30–90	Active
Terra/Advanced Spaceborne Thermal Emission and ReflectionRadiometer (ASTER)	1999	15–90	Active
Terra/Moderate-Resolution Imaging Spectroradiometer (MODIS)	1999	250–500	Active

a Resolutions are noted for the multispectral bands. Satellite information includes multiple versions (i.e. Landsat 5, Landsat 8).

b Not a satellite but is included here due to its use in some conservation studies.

Of the 42 studies that classified land cover from satellite imagery, 28.6% solely performed habitat classification, while 59.5% also calculated land cover change and/or patch-based metrics, and 11.9% also calculated vegetation height, primary productivity, or soil moisture, or identified invasive species occurrence. Of 10 studies examining land cover change since 1990 or earlier, 90% used Landsat (launched in 1972 as Earth Resources Technology Satellite) and 10% used SPOT (launched in 1986) (Table 2). Of 33 studies quantifying recent land cover (since 2000), 61.8% used imagery ≥30 m resolution, and 37.5% did so to quantify small geographic areas (≤100,000 ha).

## Discussion

Differences in satellite imagery resolution are not trivial, and can manifest into stark differences in land cover classifications and resulting patch-level metrics (i.e. habitat size, shape, and connectivity). Ultimately these discrepancies are likely to influence interpretations of fragmentation patterns of a landscape, which can directly impact species and ecosystem modeling and conservation management plans. Many types of satellite imagery are available to conservation practitioners, but based on our literature review, most current conservation research does not take full advantage of either high-resolution or low-resolution imagery. Although high-resolution imagery can be difficult to obtain, primarily due to cost [5], we found that such imagery is critical for the detection of small, narrow forest fragments (Fig. 4). Mapping small, linear habitat features, such as riparian corridors, and potential stepping-stone patches is critical to ecological studies, as these features may serve important roles in landscape connectivity [64], [65]. Furthermore, high-resolution imagery could greatly aid in the refined detection of forest loss and in the design and monitoring of potential biological corridors (e.g. Mbaracayú-San Rafael conservation corridor [66]). In Paraguay, forest loss can be dramatic, yet much of the monitoring of such loss is done primarily with Landsat data [48], [49], [67], [68]. Access to high-resolution imagery is invaluable and timely, given the ongoing and rapid deforestation in Paraguay [67], [68], and elsewhere globally [20].

Our review of current articles in *Biological Conservation*, *Biotropica*, and *Conservation Biology* revealed that more than 70% of studies quantifying land cover used previously published material, aerial photos, paper maps, or Google Earth as their main resources, instead of satellite imagery (of any resolution). When satellite imagery was used, Landsat (30 m resolution) was most common (Fig. 5a). Although Landsat imagery is important in its historical longevity and can be appropriately used to assess large geographic regions and coarse-scale phenomena, we found that studies classifying recent (since 2000) land cover of smaller areas (<100,000 ha) still relied primarily on coarse imagery (i.e. ≥30 m resolution) (Fig. 5b). High-resolution imagery has thus not been used to its full extent in conservation, yet the differences in classification and resulting landscape and patch metrics could be critical for land cover assessments. For example, Rosa et al. [69], found that 73% of the deforestation in the Brazilian Amazon in 2009 was the result of small clearings (<50 ha), thus, may go undetected by regional assessments commonly based on low resolution imagery.

Our literature review aimed to highlight the application of high-resolution imagery in studies published in journals that target conservation biologists. We found the usage of high-resolution imagery to be minimal. Moreover, most studies used previously published data sets, and some were found to use land cover data from >14 years prior to the field data collection. Although conservation biologists are not limited to these journals, conservation practitioners may not be reached by the remote-sensing, physical geography, informatics, and ecology journals that often focus on the applications of satellite imagery (see reviews by [2], [4], [36]–[44]). Furthermore, these journals do not necessarily focus on conservation applications, and therefore the utility and relevance of high-resolution imagery may be missed by conservation scientists. Even within the field of ecology, there has been a disconnect between ecologists, who may not fully utilize imagery because of the perception that it is useful at only relatively coarse spatial scales, and remote sensing specialists who tend to focus on technological issues of remote-sensing applications [36]. With increasing availability of higher-resolution imagery, however, spatial scale is no longer a limiting factor. Our findings reinforce that high-resolution imagery is important for conservation applications, but that many conservation biologists are not taking full advantage of this resource.

Our results highlight that small or narrow patches in a landscape may fail to be delineated with coarser imagery. Although the total area of a particular land cover class (e.g., forest) may not substantially increase, higher-resolution imagery is more likely to discern small and linear habitat features, which may be vital to landscape connectivity. Given that human activities have caused the conversion of more than 50% of the world’s terrestrial surface [70], most landscapes have lost the vast majority of their historic, native habitat. Such habitat loss necessitates our ability to design conservation plans and strategies that are able to reconnect or improve the functionality of remaining land cover [71]. For example, in the human-modified landscapes of Paraguay, narrow forest patches may serve as valuable corridors between core habitat areas (as outlined in [66]) for megafauna (e.g. jaguar, *Panthera onca* [72]; tapir, *Tapirus terrestris* [73]), medium-sized rare mesocarnivores (e.g. bushdog, *Speothos venaticus* [74]; oncilla, *Leopardus tigrinus* [75]; margay, *Leopardus wiedii* [75]), or arboreal species (e.g. howler monkey, *Alouatta caraya* [76]; capuchin monkey, *Sapajus cay* [77]). They may also provide important conduits of movement for pollinators and seed dispersers, which help ensure ecosystem functioning and forest regeneration and succession [78]–[81]. Therefore, research linking remotely-sensed land use change with species movement and persistence is particularly important in Paraguay given that much of what is known regarding the country’s fauna is from approximate distributions or preliminary field data [82]. Recent and noteworthy species records for Paraguay [83], including records of rodent [82], [84], [85], and bat [86] species in the Interior Atlantic Forest, exemplify the need for further field studies in the region. We propose that future conservation studies would be enhanced by access to low-cost, high-resolution imagery.

Additionally, high-resolution imagery will be valuable for more precise evaluation of habitat area and edge in landscapes. While species-area relationships have been widely used for conservation [87]–[92], improved imagery will help to better understand the effects of patch area, edge, shape, and configuration, as well as the matrix, on biodiversity. These patterns are species-specific, may vary across systems, and are often complex [93]–[97]. In Paraguay, for example, small mammal diversity increased toward the edges of large forest remnants [98]; therefore high-resolution imagery could help in precisely defining these edges and any area-to-edge (shape) relationships.

Classification using IKONOS imagery improved the delineation of forest fragments, with smaller mean size than fragments delineated using Landsat imagery. These differences were not surprising, however, given the smaller pixel size of IKONOS. Unexpectedly, however, the IKONOS-based classification led to a delineation of fewer forest patches (229) than did Landsat (237 patches) for patches ≥0.5 ha. While a difference of 8 patches may seem trivial, a classification using higher resolution imagery is expected to result in a greater number of patches (not less), due to a greater ability to distinguish smaller features. In contrast, we found that Landsat missed detecting several small forest fragments and narrow riparian corridors that connected other habitat patches, thus resulting in a classification of more disjunct, parcellated patches instead of one larger, interconnected patch. These findings have important consequences when evaluating fragmentation effects, especially in the case of heterogeneous and complex landscapes where habitat patches may be irregular and/or linear in shape, and where corridors may be critical for the movement of individuals and populations of a variety of species.

Although our findings are site-specific to eastern Paraguay, like Masuoka et al. [27], we found that Landsat classified total areas of common cover types comparable to IKONOS but the latter is only able to capture smaller habitat patches. Similarly, Kennedy [30], found that Landsat data (unlike IKONOS) failed to capture the majority of native forest patches within the karst countryside in central Jamaica, which is characterized by small forested hilltops, often <10 ha in size, but that support extensive native bird assemblages in the region [99] as well as maintain essential landscape-level connectivity (inter-patch recolonization) [100]. Landsat has also been found to insufficiently quantify selective logging due to its low resolution [7]. These findings stress the importance of using high-resolution imagery to monitor land cover change, classify small, linear, or irregularly-shaped remnants, detect narrow corridors, and delineate areas of disturbance within a larger habitat patches. Many of the protected areas and reserves in eastern Paraguay are relatively small in size: Reserva Ecológica de Capiibary (3,082 ha), Monumento Natural Macizo Acahay (2,500 ha), Reserva para Parque Nacional Ñacunday (2,000 ha), and Monumento Científico Moisés Bertoni (199 ha). Yet, despite their size, we would expect these areas to provide critical refugia for Atlantic Forest species, and increasingly so if current rates and patterns of land-cover transformation continue. Thus, being able to effectively monitor fine-scale habitat changes in these important yet small reserves and parks has increasing conservation importance in Paraguay (e.g. via management projects like Paraguay Biodiversidad, [101]), as well as in other tropical regions undergoing rapid change [20].

Although free data sets and free satellite imagery are available online [40], many of these resources are at lower resolutions (e.g. MODIS, Landsat), limited by the dates available (e.g. 4-m multispectral OrbView-3 data are available for free, but only from 2003–2007 without full global coverage) and/or not modifiable (e.g. Google Earth). When using lower-resolution imagery to analyze land cover without field verification of the modeling results, evaluations of connectivity and appropriate corridors may be incorrect and/or misleading [102]. We agree with Arponen et al. [103], that analyses using high-resolution data are feasible and important, especially when assessing connectivity of habitat. Alongside the need to match spatial resolution appropriately with spatial problems, we emphasize the need to use an appropriate temporal scale and to be cautious in extrapolating from imagery and land cover data that are temporally distant from field data. Access to a variety of imagery dates is also important as seasonality can impact land cover classification accuracy [104], and land cover change can occur quickly.

High-resolution imagery, as well as long-term, time-series data (e.g. Landsat), are essential for conservation, economic, and social research and applications [52]. Although various governments and private satellite companies (e.g. GeoEye, DigitalGlobe) have provided much-needed access to satellite imagery for environmental research, access to these products should increase [17], [20]. A recent call by Lynch et al. [105] to governments to use satellites for monitoring illegal logging highlights the importance of this issue. Habitat conversion can happen quickly and covertly [106], and at times illegally; therefore, high-resolution imagery needs to be available for regular ecosystem monitoring. Information derived from satellite imagery should also be shared in user-friendly formats with environmental policy groups [1], as well as conservation and education leaders. To better ensure the prevalence of satellite image analyses for conservation purposes, we recommend increasing interdisciplinary training and collaboration among conservation, environmental, and remote-sensing fields [4], [13], and improving the accessibility to high-resolution imagery at low (or no) cost.
